# The association between psychological distress and alcohol consumption and physical activity: a population-based cohort study

**DOI:** 10.3389/fpsyt.2023.1181046

**Published:** 2023-06-22

**Authors:** Silvia Eiken Alpers, Ståle Pallesen, Jørn Henrik Vold, Ellen Haug, Linn-Heidi Lunde, Jens Christoffer Skogen, Asgeir Mamen, Silje Mæland, Lars Thore Fadnes

**Affiliations:** ^1^Department of Addiction Medicine, Haukeland University Hospital, Bergen, Norway; ^2^Department of Clinical Psychology, Faculty of Psychology, University of Bergen, Bergen, Norway; ^3^Department of Psychosocial Science, Faculty of Psychology, University of Bergen, Bergen, Norway; ^4^Optentia Research Focus Area, North-West University, Vanderbijlpark, South Africa; ^5^Bergen Addiction Research, Department of Addiction Medicine, Haukeland University Hospital, Bergen, Norway; ^6^Department of Global Public Health and Primary Care, Faculty of Medicine, University of Bergen, Bergen, Norway; ^7^Division of Psychiatry, Haukeland University Hospital, Bergen, Norway; ^8^Department of Health Promotion and Development, Faculty of Psychology, University of Bergen, Bergen, Norway; ^9^Department of Teacher Education, NLA University College, Bergen, Norway; ^10^Department of Health Promotion, Norwegian Institute of Public Health, Bergen, Norway; ^11^Alcohol and Drug Research Western Norway, Stavanger University Hospital, Stavanger, Norway; ^12^Centre for Evaluation of Public Health Measures, Norwegian Institute of Public Health, Oslo, Norway; ^13^School of Health Sciences, Kristiania University College, Oslo, Norway

**Keywords:** alcohol consumption, physical activity, psychological distress, worries, risk factors, pandemic (COVID-19)

## Abstract

**Introduction:**

The COVID-19 pandemic and infection control measures caused changes to daily life for most people. Heavy alcohol consumption and physical inactivity are two important behavioral risk factors for noncommunicable diseases worldwide. The COVID-19 pandemic, with its social distancing measures, home office policies, isolation, and quarantine requirements may have an impact on these factors. This three-wave longitudinal study aims to investigate if psychological distress and worries related to health and economy were associated with levels and changes in alcohol consumption and physical activity during the two first years of the COVID-19 pandemic in Norway.

**Methods:**

We used data collected in April 2020, January 2021, and January 2022 from an online longitudinal population-based survey. Alcohol consumption and physical activity status were assessed at all three measuring points *via* the Alcohol Use Disorder Identification Test (AUDIT-C) and the International Physical Activity Questionnaire (IPAQ-SF). COVID-19-related worries, home office/study, occupational situation, age, gender, children below 18 years living at home, and psychological distress (measured with the Symptom Checklist (SCL-10)) were included as independent variables in the model. A mixed model regression was used and presented with coefficients with 95% confidence intervals (CI).

**Results:**

Analysis of data from 25,708 participants demonstrates that participants with substantial symptoms of psychological distress more often reported higher alcohol consumption (1.86 units/week, CI 1.48–2.24) and lower levels of physical activity [−1,043 Metabolic Equivalents of Task (METs) per week, CI −1,257;−828] at baseline. Working/studying from home (0.37 units/week, CI 0.24–0.50) and being male (1.57 units/week, CI 1.45–1.69) were associated with higher alcohol consumption. Working/studying from home (−536 METs/week, CI −609;−463), and being older than 70 years (−503 METs/week, CI −650;−355) were related to lower levels of physical activity. The differences in activity levels between those with the highest and lowest levels of psychological distress reduced over time (239 METs/week, CI 67;412), and similarly the differences in alcohol intake reduced over time among those having and not having children < 18 years (0.10 units/week, CI 0.01–0.19).

**Conclusion:**

These findings highlight the substantial increases in risks related to inactivity and alcohol consumption among those with high levels of psychological distress symptoms, and particularly during the COVID-19 pandemic, and increase the understanding of factors associated with worries and health behavior.

## 1. Introduction

Harmful alcohol consumption and physical inactivity are two of the most important behavioral risk factors for noncommunicable diseases (1). These risk factors are also associated with mental health problems (2–5). Additionally, an association between harmful drinking and physical inactivity has been found (6–8). Drinking above the recommended limits increases the risk of developing health problems such as liver disease, hypertension, stroke, heart disease, and several cancers (9, 10). It can also lead to an increased risk of falls and is associated with a higher risk of depression, anxiety, and other mental health problems (11, 12). Alcohol can have harmful effects on the nervous system, including impaired cognitive function and motor coordination. It affects the brain’s ability to process information and make decisions, which can lead to poor judgment and increased reaction times (13). This can increase the risk of accidents and injuries, particularly those involving driving, operating machinery, or engaging in other activities that require focus and coordination (14, 15). Older adults may also experience decreased medication effectiveness because of alcohol use (14) or adverse medication-alcohol interactions. Concomitant use of prescription drugs such as benzodiazepines and opioids, which many older people use, can increase the risk of side effects of the drugs and the negative effects of alcohol (16). Increased reaction times, impaired coordination, and decreased physical ability can also affect a person’s motivation and ability to participate in physical activity (PA) (17, 18). It is well known that PA offers several health benefits (19–23) and can positively impact alcohol behaviors (24, 25). Regular PA has been associated with lower levels of stress, anxiety, and depression (26). Engaging in PA can help improve mood, boost self-esteem, promote relaxation, and enhance overall mental well-being (19). Harmful alcohol consumption and persistent physical inactivity, on the other hand, negatively affect overall life expectancy and increase the prevalence of chronic diseases (27–29).

The pandemic caused by the coronavirus disease 2019 (COVID-19) had a significant impact on the daily lives of most people (30). It is likely that this global health crisis had a profound effect on the physical and mental health, as well as the overall well-being, of the general population (31, 32). Moreover, lifestyle behaviors, including alcohol consumption, were also affected by the pandemic (33). In terms of the pandemic, systematic reviews (34–36) display heterogenic results regarding alcohol consumption across countries and regions, a decrease was reported in some countries (e.g., Australia, Germany, Norway), an increase was found in other countries (e.g., New Zealand, Ireland, Canada) while no change was reported in yet some other countries (e.g., United Kingdom, Finland, Belgium). During the first wave of COVID-19, alcohol consumption predominantly declined in Europe (35). Mitigation of alcohol control measures and growing personal distress related to the COVID-19 outbreak could still lead to an increase in alcohol consumption long-term (37, 38).

A growing literature shows that people with mental health problems may be especially vulnerable to increased drinking during a pandemic (39, 40). Both acute and chronic stress are documented risk factors for increased alcohol use in general (41–43). Hence, increased alcohol use can be regarded as a response to a crisis as well as a coping mechanism to relieve stress (44–46). Infection control measures, like physical or social distancing, have been found to lead to loneliness, lower life satisfaction, and increased mental health problems, which in turn can cause higher alcohol use (47–49). While the mental health effects of the pandemic may have led people to drink alcohol more frequently to cope with stress and anxiety, it is also possible that the closure of social spaces where drinking usually takes place has resulted in a decrease in social drinking (50). In addition, the crisis caused by the COVID-19 pandemic may have led some people to abstain from alcohol due to the uncertainty and fear it caused (51, 52). Abstinence from alcohol may have been a way for some to maintain a sense of control and stability during a time when other aspects of life seemed uncontrollable (42). The pandemic may have also affected drinking behavior through the influence of health and financial concerns (53). On the other hand, studies on the effects of the COVID-19 pandemic have attested to a shift in the location of alcohol consumption from bars and restaurants to homes, leading to a rise in the frequency of alcohol consumption at home compared to before the pandemic (36, 54).

The COVID-19 pandemic had a significant impact on people’s daily PA levels (55, 56). With many parts of the world under lockdown, typical activities such as going to the gym and participating in sports were no longer possible. Moreover, the use of home offices led to a reduction in PA related to commuting to and from work (e.g., cycling/walking to work, walking to and from public transport). Physical inactivity and daily sitting time increased during the quarantine period, especially among the elderly (56, 57). Some countries also put restraints on PA by enforcing curfews as a disease-control measure (58). Despite restraints on PA, various resources were available to help people stay active during the pandemic. Exercises such as bodyweight workouts, running, and online and outdoor group classes were encouraged and many countries, like Norway, never resorted to curfews to control COVID-19 (59). Hence, in most countries, people could take advantage of their natural environment and participate in outdoor PA while adhering to social distancing guidelines (60).

Most of the aforementioned studies on the impact of the pandemic on mental health, alcohol use, and PA mainly covered the early stages of COVID-19. Therefore, more knowledge is needed to elucidate the whole period from the early phases of the COVID-19 pandemic (spring of 2020) to the late phases when restrictions ceased in 2022. We opted to include control variables for home office/study and temporary layoffs, based on previous findings from our research group regarding the relationship between pandemic-related measures and alcohol consumption during periods of lockdown (61). Against this backdrop, the aim of this longitudinal cohort study was to assess associations between psychological distress and worries related to health and economy, and to changes in alcohol consumption during the two first years of the COVID-19 pandemic. We also investigate associations between psychological distress and worries related to health and economy with alterations in PA.

## 2. Materials and methods

### 2.1. Data source

**Figure 1:**
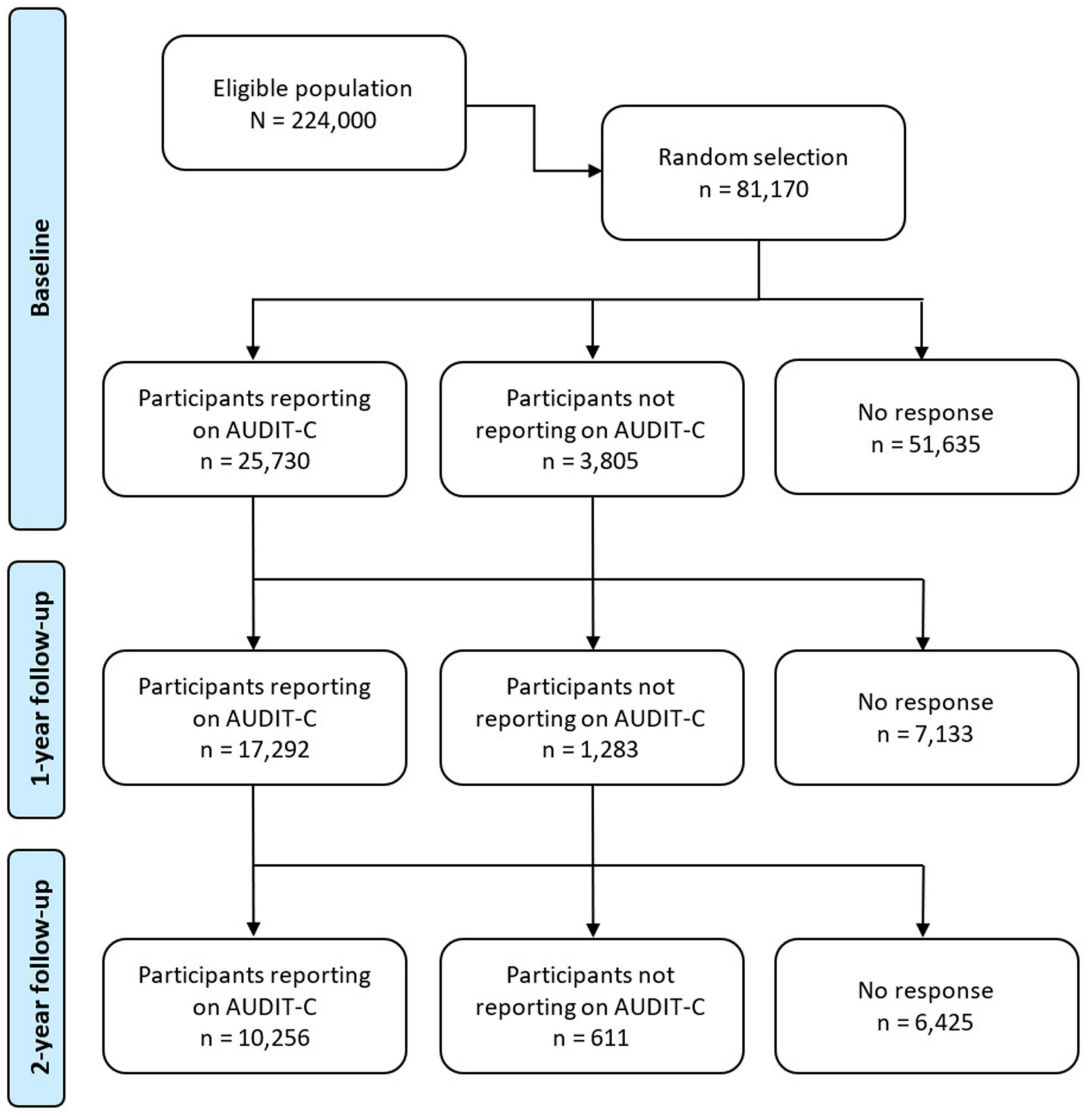
Flow chart of the study design and cohort overview. Schematic of the longitudinal study design including wave 1, which consisted of 2 weeks of baseline assessment (T0), wave 2 after 9 months covering 4 weeks of data collection (T1), and wave 3 after 12 months again covering 4 weeks of data collection (T2).

In April 2020, we invited a representative sample of 81,170 people from a total of 224,000 adult inhabitants in the city of Bergen in Western Norway to participate in the study “Bergen in Change” (BiE-study) surveying the potential impact of the lockdown on everyday life, health, and health behaviors during the COVID-19 pandemic (62). The sample was selected randomly and matched the general population in terms of age and sex. People invited to participate were identified using the National Population Registry of Norway and the common contact register^1^. In total, 29,535 people (response rate 36%) consented to participate in the present study at the first wave (T0; Figure 1).

### 2.2. Data collection

A set of electronic questionnaires was distributed *via* email and short text messages (SMS) using the online data collection tool SurveyXact. The questionnaire included demographic information and questions about various aspects of life and health related to the COVID-19 pandemic and lockdown. The first data collection (T0) took place between April 15 and April 30, 2020. One month before T0, several restrictions (social distancing, closure of educational, cultural, and training/sport/gym facilities, requirements to work from home, and introduction of quarantine requirements) due to COVID-19 had been initiated.

All who participated at T0 were invited to respond to the survey at T1 where the data collection took place between December 2020 and January 2021. By this time, the restrictions had been eased slightly. Schools had reopened, and organized sports activities were slowly picking up. Restaurants and cafes were allowed to open with limited capacity. However, social distancing and the advice to avoid public transport were still maintained (63). In January 2021, restrictions were re-imposed in response to a new wave of the virus. At T1, 18,575 people participated (response rate 63% from T0), with a median time interval of 9 months between the first data collection and the follow-up assessment.

The third wave of data collection (T2) took place between December 2021 and January 2022, roughly coinciding with the end of the pandemic measures. Most of the restrictions put in place in April 2020 were lifted. Despite the easing of restrictions, people were still urged to practice social distancing and to wear face masks in shops and public institutions if it was not possible to keep a one-meter distance. The median time interval between the second and the third data collection was 12 months. In all, 10,867 (response rate 37% from T0) persons participated in the third wave.

### 2.3. Measures

The main outcome variables in the present study were self-assessed alcohol consumption and PA. Alcohol consumption was assessed by the short version of the Alcohol Use Disorders Identification Test Consumption (AUDIT-C), which consists of the first three questions of the full AUDIT (64, 65). The three questions investigate the frequency of drinking, typical quantity consumed, and frequency of heavy drinking: 1. How often did you have a drink containing alcohol (in the past year)? 2. How many units of alcohol do you drink on a typical day when you are drinking? 3. How often do you drink 6 or more units of alcohol on the same occasion? Each question is scored using a five-point scale ranging from 0 to 4; thus, the composite score of the AUDIT-C ranges from 0 to 12. In the present study, we collapsed AUDIT-C scores into five categories (0–2 = low risk, 3–5 = moderate risk, 6–7 = high risk, and 8–12 = severe risk drinking for women and 0–3 = low risk, 4–5 = moderate risk, 6–7 = high risk, and 8–12 = severe risk drinking for men) for ease of presentation and to ensure adequate precision in estimating the severity of problem drinking in each group. The cut-offs were aligned with previously demonstrated criteria or predictive validity (66, 67). At all three measuring points, about 91% of the participants reported consumption of alcohol.

The short form of the International Physical Activity Questionnaire (IPAQ-SF) (68) was used to collect data on the level of PA. The questions of the IPAQ-SF allowed measuring the total weekly PA energy expenditure of the participants (i.e., the sum of walking, moderate-intensity PAs, and vigorous-intensity PAs) in terms of Metabolic Equivalent Task minutes per week (METs/week). According to the IPAQ-SF scoring guidelines^2^, high PA is equivalent to >1 h of moderate-intensity activity over and above basal activity or > 30 min of vigorous-intensity activity above basal levels daily. Moderate activity is defined as 30 min of at least moderate-intensity activity on most days of the week. Low activity describes all subjects not meeting the two criteria described above. Participants were accordingly categorized into these three PA classes (low, moderate, and high). At baseline (T0), 93% of the participants reported their PA levels. The response rate dropped to 87% at T1 and 88% at T2, respectively. Exposure variables were psychological distress, COVID-19-related worries, and lockdown consequences of pandemic measures. The items being temporarily laid-off, and having home office/studying from home, were designed as dichotomous nominal variables and formulated as true/false statements. The questionnaire included two questions on economic worries: 1. “I fear (am worried) that the outbreak will cause me to be laid off or lose my job.” 2. “I fear (am worried) that the outbreak will lead to a worsening of my economic situation.” The responses were recorded on a three-point scale with the response alternatives “strongly agree,” “agree,” and “disagree.” Categorization of “economic worries” was based on answering at least one of the two questions with “strongly agree.” The “health worries” variable reflected how COVID-19 may affect one’s or others’ health: 1. “I have become scared and anxious (worried) that the infection will affect some of my loved ones.” 2. “I have become scared and anxious (worried) that the infection will affect me.” 3. “I have become scared and anxious (worried) that the infection will affect some of the elderly members of the family.” For each of these items the respondents were to indicate their level of agreement by choosing one of three responses (“strongly agree,” “agree,” and “disagree”). We defined “health worries” as answering at least one of the questions with “strongly agree,” which was assigned a score of 1; otherwise, the score was set to 0. For more detailed information see Alpers et al. (61).

Psychological distress was measured with the Norwegian-validated translation of the ten-item version of the Hopkins Symptom Checklist (SCL-10) (69). The participants rated how frequently they had experienced symptoms of anxiety and depression during the past 7 days on a 4-point Likert scale, ranging from 1 (not at all) to 4 (extremely). The mean score of all items was used as the measure of psychological distress. A mean score index was derived from the SCL-10 scale and was compressed to a 0–1 continuous scale with 0 indicating no psychological distress and 1 indicating maximum (severe) psychological distress. We also created a dichotomous variable for having an average score above 1.85 in the full-length score (1–4), which is considered a valid cut-off value for the prediction of significant psychological distress (69). Approximately 95% of the participants had a valid SCL-10 score at all three measurement points.

Covariates, such as gender, age, and children < 18 years at home, were measured at baseline.

### 2.4. Statistical analyses

Stata/SE 17.0 (StataCorp, College Station, TX, United States) was used for all descriptive and regression model analyses. Sankeymatic^3^ was used to generate Sankey diagrams for a graphical presentation of the changes in alcohol use and PA over time. The threshold for statistical significance was set to *p* < 0.05 for all analyses unless otherwise stated. In all the analyses, time was defined as the number of years from baseline.

Weighted estimates for the exposure variables are presented with their corresponding 95% confidence intervals (CI). Chi-square tests were used to test for statistically significant differences between groups of categorical variables.

Linear mixed model analyses were used to investigate whether the exposure variables were associated with drinking behavior and PA at baseline and the extent to which they influenced any changes in the drinking behavior and PA from baseline (T0) to the following (T1 and T2) measuring points one and 2 years later. We based the analyses in this paper on questionnaires that had valid responses to all questions in AUDIT-C (*n* = 25,708; 87% of the total sample). The exposure variables were kept constant at the baseline level in predicting the level and changes in the outcome variables. To explore whether exposure variables predicted changes in outcomes, interactions between these variables and time were added to the model. Maximum Likelihood estimation was used. All available responses to the outcome variables were included in the analyses.

### 2.5. Ethics approval and consent to participate

The participants provided informed consent to participate before answering the questionnaires. They were also guaranteed confidentiality and the right to withdraw from participation. The project was approved by the Regional Committee for Medical and Health Research Ethics, Health Region West (ethics registration code 2020/131560). It was conducted in accordance with guidance from data protection officials at the University of Bergen.

## 3. Results

### 3.1. Study sample

At baseline, the median age of the participants was 50 years (interquartile range (IQR) 36–63), 56% were women, 40% had more than 3 years of university or college as their educational attainment, 94% were Norwegian citizens, 87% had a household-adjusted income above 25,000 euros (EUR 1 ≈ Norwegian krone (NOK) 10) per person, 68% were employed/worked, and 8% were students (Table 1). Two-thirds lived together with 1–3 other people.

**Table 1:** Background characteristics of participants at baseline and follow-up.

Total	Baseline *n* (%)	1-year follow-up *n* (%)	2-year follow-up *n* (%)
*n*	25,708 (100%)	17,292 (100%)	10,256 (100%)
Gender (women)	14,452 (56%)	9,749 (56%)	5,886 (57%)
Primary school	1924 (8%)	1,073 (7%)	598 (6%)
High school	7,246 (28%)	4,404 (27%)	2,542 (26%)
University ≤ 3 years	6,157 (24%)	3,950 (24%)	2,332 (24%)
University > 3 years	10,246 (40%)	6,874 (42%)	4,315 (44%)
Adjusted income (EUR)^*^
0–25,000	3,080 (13%)	1,589 (11%)	877 (10%)
25,000–50,000	10,051 (44%)	6,346 (43%)	3,799 (43%)
> 50,000	9,789 (43%)	6,797 (46%)	4,199 (47%)
Persons in household
1	5,182 (21%)	3,513 (22%)	2,186 (23%)
2	8,057 (32%)	5,436 (34%)	3,377 (35%)
3–4	8,776 (35%)	5,265 (33%)	3,077 (32%)
5+	2,935 (12%)	1708 (11%)	934 (10%)
Employment	17,447 (68%)	10,939 (63%)	6,468 (63%)
Student/school	2011 (8%)	840 (5%)	456 (4%)
Placed in quarantine	4,173 (16%)	2,808 (16%)	1,626 (16%)
Temporarily laid-off	1940 (8%)	1,076 (6%)	581 (6%)
Home office/study	12,646 (49%)	8,276 (48%)	4,832 (47%)
COVID-19 symptoms	1,581 (6%)	985 (6%)	594 (6%)
Worries	13,081 (51%)	7,964 (46%)	4,609 (45%)
Worries related to economy	4,179 (16%)	2,299 (13%)	1,271 (12%)
Health-related worries	11,367 (44%)	6,981 (40%)	4,051 (40%)
Psychological distress	5,052 (20%)	2,888 (17%)	1,672 (16%)

* The adjusted income is the household income divided by the personal index. The personal index is calculated as 1 for the first adult, 0.7 per other adult household member, and 0.5 per child. The adjusted income was converted to Euros.

Tables for background characteristics of participants per age group at baseline, 1-year follow-up, and 2-year follow-up separately are available in the Supplementary Tables 1–3.

### 3.2. Alcohol consumption

**Figure 2:**
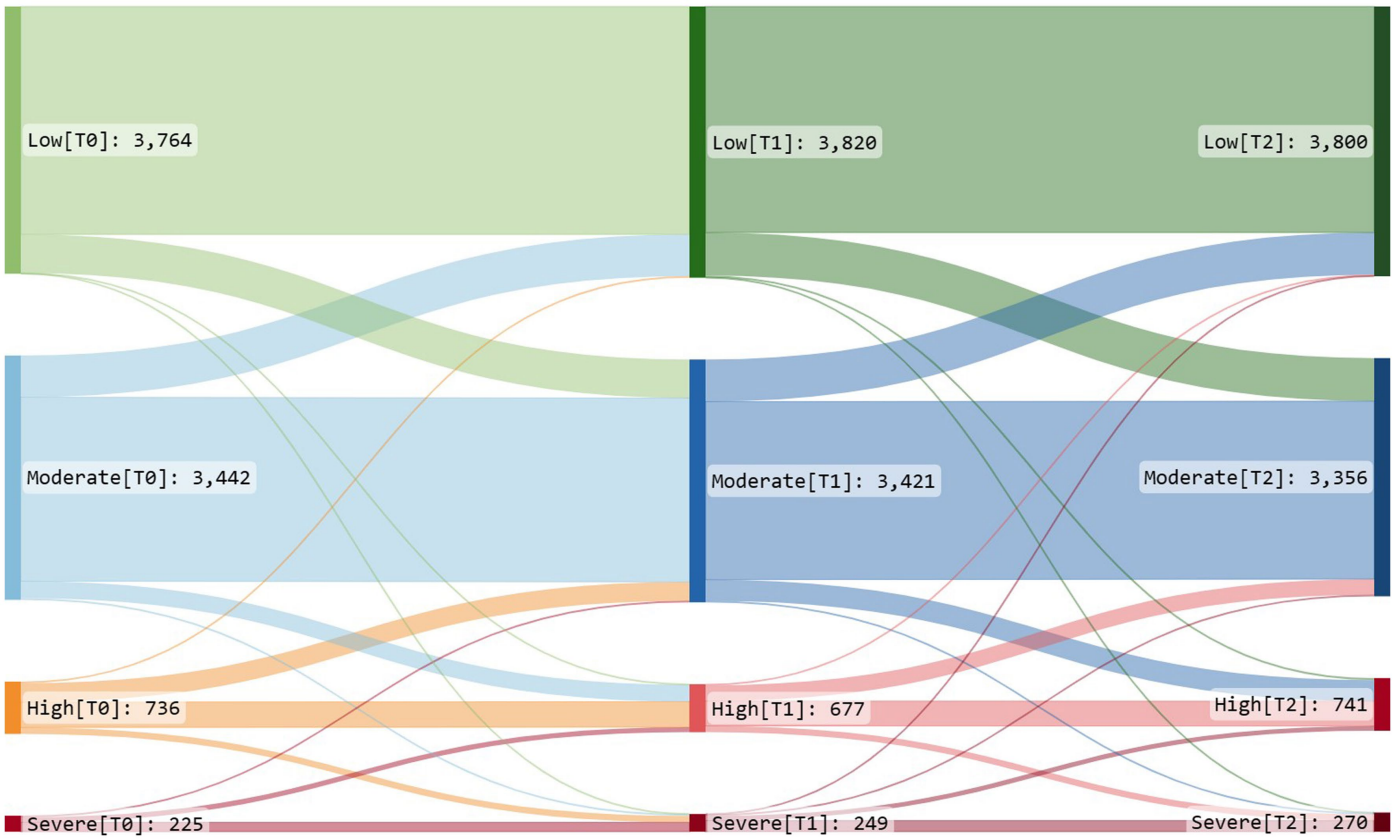
Sankey diagram of change in alcohol consumption. Low = AUDIT-C score ≤ 2 for women and ≤ 3 for men. Moderate = AUDIT-C score > 2 and ≤ 5 for women and > 3 and ≤ 5 for men. High = AUDIT-C score > 5 and ≤ 7 for women and men. Severe = AUDIT-C score ≥ 8 for women and men. The diagram shows alcohol consumption broken down into four levels (low, moderate, high, and severe) at three time points (T0, T1, and T2). The connecting paths show the proportion of individuals changing or not changing alcohol consumption levels across the time points. The width of each path represents the proportion of individuals who change category. The colors highlight the different levels of risk associated with alcohol consumption and make it easier to compare the relative risk levels across different categories. Green is used to represent low-risk drinking levels, blue for moderate-risk levels, orange for high-risk levels, and red for severe-risk drinking levels, respectively. Sankey diagrams of change in alcohol consumption per gender are available in the Supplementary Figures 1, 2.

A total of 13% of the participants reported use of alcohol above the cut-off score for high-risk drinking at T0 according to the AUDIT-C (Table 2). The low-risk, moderate-risk, and high-risk drinking levels remain almost unchanged over the 2 years (Figure 2). The severe-risk drinking level, on the other hand, increases by 20% from T0 to T2. The group that reported severe psychological distress had higher levels of alcohol consumption, roughly two units more per week, than those with no distress (coefficient: 1.86, 95% CI: 1.48;2.24). This difference was sustained over the 2 years.

**Table 2:** Drinking behavior and physical activity (PA) levels in relation to age at baseline, 1-year follow-up, and 2-year follow-up [*n* (%)].

Age	18–29	30–39	40–49	50–59	60–69	70+	Total
Baseline							
Low-risk drinking^*^	1,064 (32%)	1,900 (46%)	2,079 (44%)	2,410 (46%)	2,108 (47%)	2,269 (63%)	11,830 (46%)
Moderate-risk drinking	1,406 (42%)	1,712 (41%)	2,073 (44%)	2,209 (42%)	1,826 (41%)	1,098 (31%)	10,324 (40%)
High-risk drinking	658 (20%)	407 (10%)	398 (8%)	489 (9%)	403 (9%)	162 (5%)	2,517 (10%)
Severe-risk drinking	210 (6%)	131 (3%)	161 (3%)	157 (3%)	142 (3%)	50 (1%)	851 (3%)
PA level low	933 (29%)	1,156 (29%)	1,151 (26%)	1,067 (22%)	808 (21%)	707 (27%)	5,822 (25%)
PA level moderate	1,377 (42%)	1727 (43%)	1931 (44%)	2,116 (44%)	1,683 (44%)	1,150 (44%)	9,984 (43%)
PA level high	944 (29%)	1,140 (28%)	1,355 (31%)	1,608 (34%)	1,346 (35%)	772 (29%)	7,165 (31%)
1-year follow-up							
Low-risk drinking^*^	454 (32%)	1,010 (44%)	1,244 (43%)	1,640 (45%)	1,555 (46%)	1,608 (62%)	7,511 (46%)
Moderate-risk drinking	633 (44%)	990 (43%)	1,348 (46%)	1,538 (43%)	1,405 (42%)	840 (32%)	6,754 (42%)
High-risk drinking	260 (18%)	236 (10%)	235 (8%)	327 (9%)	309 (9%)	124 (5%)	1,491 (9%)
Severe-risk drinking	86 (6%)	76 (3%)	88 (3%)	100 (3%)	97 (3%)	38 (1%)	485 (3%)
PA level low	422 (29%)	666 (29%)	729 (26%)	729 (22%)	589 (20%)	477 (24%)	3,612 (24%)
PA level moderate	629 (43%)	1,030 (44%)	1,275 (45%)	1,517 (45%)	1,339 (45%)	911 (46%)	6,701 (45%)
PA level high	398 (27%)	641 (27%)	855 (30%)	1,115 (33%)	1,039 (35%)	599 (30%)	4,647 (31%)
2-year follow-up							
Low-risk drinking^*^	228 (31%)	562 (45%)	712 (43%)	1,032 (46%)	1,020 (47%)	1,002 (60%)	4,556 (47%)
Moderate-risk drinking	332 (45%)	535 (42%)	749 (45%)	952 (42%)	921 (42%)	549 (33%)	4,038 (41%)
High-risk drinking	135 (18%)	122 (10%)	136 (8%)	205 (9%)	201 (9%)	89 (5%)	888 (9%)
Severe-risk drinking	42 (6%)	41 (3%)	55 (3%)	60 (3%)	51 (2%)	27 (2%)	276 (3%)
PA level low	227 (31%)	357 (28%)	440 (27%)	453 (21%)	387 (20%)	309 (24%)	2,173 (24%)
PA level moderate	329 (44%)	573 (45%)	710 (44%)	971 (46%)	884 (45%)	609 (46%)	4,076 (45%)
PA level high	185 (25%)	341 (27%)	472 (29%)	693 (33%)	688 (35%)	392 (30%)	2,771 (31%)

The table displays population-weighted estimates (age, gender, education) for percentages.

* Drinking categories are based on AUDIT-C scores.

High and severe-risk drinking was most prevalent among the youngest age group (18–29 years) and the least prevalent among the oldest age group (> 70 years). More men than women reported use of alcohol above the cut-off score for high-risk drinking at all measuring points (Supplementary Tables 4, 5).

Men drank almost twice as much as women at baseline [1.57 (CI: 1.45;1.69) Table 3]. Those from ≥ 60 to < 70 years of age had the highest alcohol consumption and consumed over half a unit more per week than the youngest age group < 30 years [0.56 (CI 0.33;0.79)]. Participants with children below 18 years at home drank one unit less per week than those without [−1.02 (CI −1.17;−0.87)]. A marginal time trend, suggesting increased consumption, appeared for the age group ≥ 50 to < 60 years [0.13 (CI 0.00;0.26)] and participants with children below 18 years at home [0.10 (CI 0.01;0.19)].

**Table 3:** Adjusted linear mixed model for the units of alcohol per week (*n* = 24,649).

	Baseline	Time trend
	Effect estimates Coefficients (95% CI)	(per year) Coefficients (95% CI)
Male	1.57 (1.45;1.69)^*^	0.04 (−0.03;0.11)
Years of age:		
18–29	(ref.)	(ref.)
30–39	−0.06 (−0.29;0.16)	−0.03 (−0.17;0.12)
40–49	0.33 (0.10; 0.55)^*^	0.03 (−0.11;0.17)
50–59	0.14 (−0.07;0.35)	0.13 (0.00;0.26)
60–69	0.56 (0.33;0.79)^*^	0.07 (−0.07;0.20)
≥ 70	0.11 (−0.15;0.36)	−0.02 (−0.17;0.13)
Children < 18 years at home	−1.02 (−1.17;−0.87)^*^	0.10 (0.01;0.19)^*^
Temporarily laid off	0.23 (−0.01;0.46)	0.04 (−0.10;0.19)
Home office/study	0.37 (0.24;0.50)^*^	−0.04 (−0.12;0.03)
Economic worries	0.14 (−0.04;0.32)	0.09 (−0.02;0.20)
Health worries	−0.23 (−0.35;−0.11)^*^	−0.03 (−0.10;0.04)
Psychological distress	1.86 (1.48;2.24)^*^	0.08 (−0.14;0.30)
Alcohol units per week	2.18 (1.95;2.40)	−0.03 (−0.17;0.11)^*^

* Significantly different from reference group (*p* < 0.05).

The table displays a linear mixed model regression analysis evaluating the associations between psychological distress and worries related to health and economy, pandemic measures, and personal situation (predictors) and adjusted for age and gender on alcohol consumption at baseline and the predictors’ influence on changes in alcohol consumption (time trend) per year from baseline. The alcohol consumption was measured by questions 1 and 2 of the AUDIT-C, ranged from 0 to 66 alcohol units per week.

CI, confidence interval.

### 3.3. Physical activity

**Figure 3:**
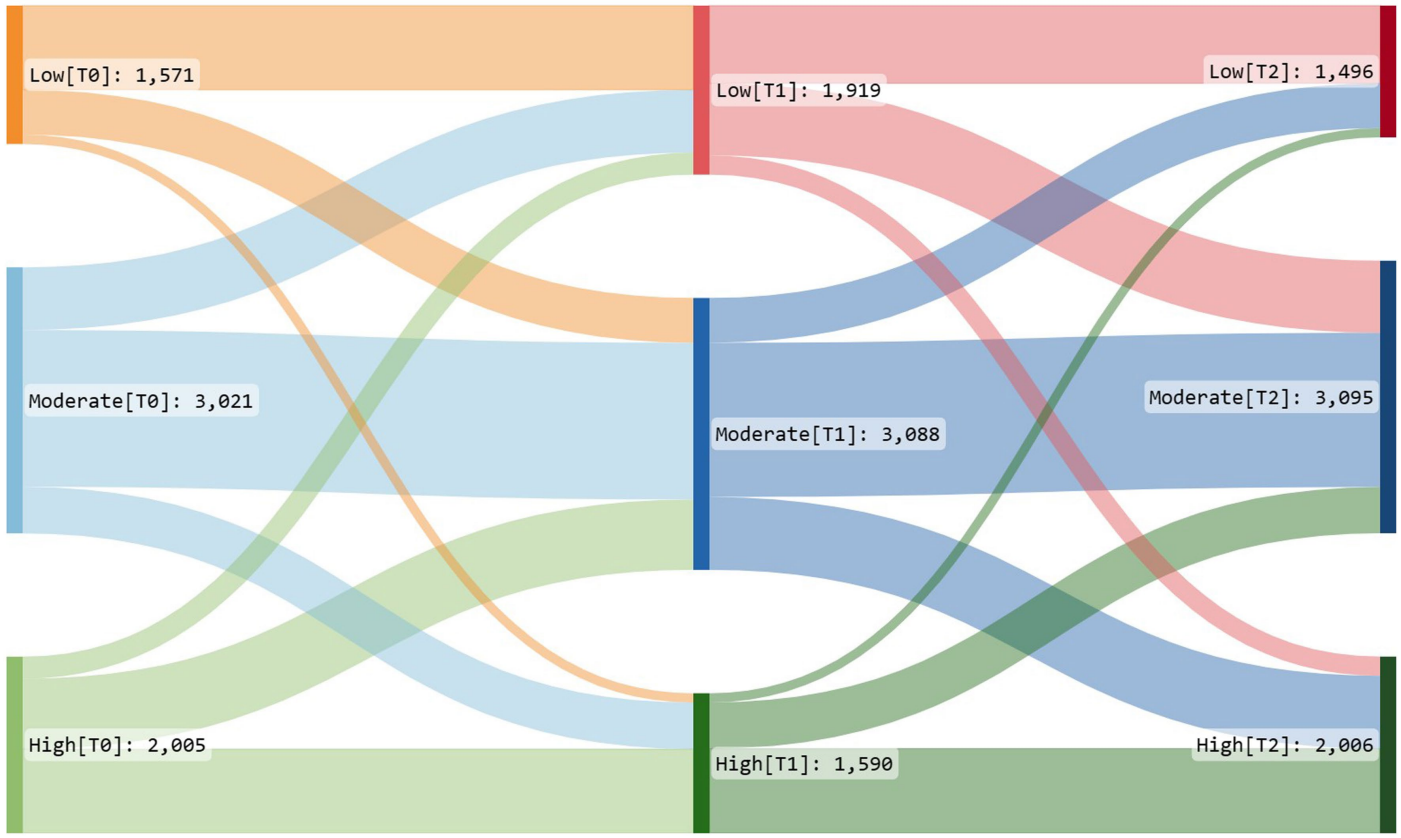
Sankey diagram of change in physical activity (PA). High = >1 h of moderate-intensity activity over and above basal activity or > 30 min of vigorous-intensity activity above basal levels daily. Moderate = 30 min of at least moderate-intensity activity on most days of the week. Low = not meeting the aforementioned criteria. The diagram shows PA broken down into three levels (low, moderate, and high) at three time points (T0, T1, and T2). The connecting paths show the proportion of individuals changing or not changing PA levels across the time points. The width of each path represents the proportion of individuals who change category. The colors highlight the different levels of PA: Red is used to represent low activity levels, blue for moderate activity levels, and green for high activity levels, respectively.

A total of 45% of the participants reported moderate PA levels at all measuring points (Table 2). However, low and high PA levels fluctuated slightly over the 2 years (Figure 3). The proportion reporting low activity levels increased 1 year into the pandemic (T1) but was close to the baseline levels 2 years into the pandemic (T2). The group that reported severe psychological distress also reported substantially less PA than those without distress [−1,043 (CI −1,257;−828)]. However, the differences in PA decreased slightly over time as those with severe psychological distress had a positive time trend in METs per week [239 (CI 67;412)].

Women had a higher proportion of change between PA levels and measuring points than men (see Supplementary Figures 3, 4). Participants over 70 years of age had the fewest METs per week compared with those under 30 years of age [−503 (CI −650;−355); Table 4]. Participants at home office/school had lower activity levels than those not working from home [−536 (CI −609;−463)].

**Table 4:** Adjusted linear mixed model for the METs per week (*N* = 23,612).

	Baseline	Time trend
	Effect estimates coefficients (95% CI)	(per year) Coefficients (95% CI)
Male	344 (275;412)^*^	16 (−38;69)
Years of age:		
18–29	(ref.)	(ref.)
30–39	−124 (−249;0)	27 (−82;136)
40–49	−74 (−200;52)	14 (−95;122)
50–59	0 (−118;118)	48 (−52;149)
60–69	−22 (−151;106)	68 (−38;174)
≥ 70	−503 (−650;−355)^*^	70 (−51;191)
Children < 18 years at home	−82 (−166;3)	−50 (−117;18)
Temporarily laid off	80 (−53;214)	−66 (−176;45)
Home office/study	−536 (−609;−463)^*^	−7 (−65;50)
Economic worries	166 (64;269)^*^	29 (−55;112)
Health worries	27 (−43;97)	−43 (−98;12)
Psychological distress	−1,043 (−1,257;−828)^*^	239 (67;412)^*^
METs per week	2,555 (2,429;2,682)	−34 (−141;74)

* Significantly different from reference group (*p* < 0.05).

The table displays a linear mixed model regression analysis evaluating the associations between psychological distress and worries related to health and economy, pandemic measures, and personal situation (predictors) and adjusted for age and gender on the METs at baseline and the predictors’ influence on changes in METs (time trend) per year from baseline.

CI, confidence interval; MET, metabolic equivalent of task.

## 4. Discussion

Substantial psychological distress was strongly associated with both substantially higher intake of alcohol and lower levels of PA. Male gender, working/studying from home, and having psychological distress were associated with increased alcohol consumption. Fewer METs per week was associated with female gender, being over 70 years old, working/studying from home, and having psychological distress. However, there was a positive time trend with reduced differences in PA levels among those with high and low levels of psychological distress. Inversely, some of the differences with lower levels of alcohol intake among participants with children under 18 years reduced over time. Economic worries were associated with higher PA levels and health worries were associated with lower levels of alcohol consumption at baseline, but no clear time trends. The results of our study showed also that home office/study was strongly associated with higher alcohol consumption and lower PA levels.

Prior research has established the relationship between psychological distress and alcohol consumption (70, 71). A recent study (72) showed that participants with higher levels of distress reported higher use of alcohol during COVID-19. Low life satisfaction and psychological distress are associated with alcohol problems (73). Disruptions to social interactions, changes in employment and parental responsibilities, and work-life balance concurrently are probably most common for those between 30 and 50 years old. They are more likely to have school-aged children and may have faced additional challenges working from home and caring for them. However, having children below 18 years of age living at home was in the present study associated with less alcohol consumption at baseline, but an increase was observed over time. This result is in contrast to a study from the United Kingdom where increased alcohol consumption was linked to living with children (74). Schools in the UK were closed for 14 weeks (75), which is 5 weeks longer than the 9-week closure period in Norway (75).

An analysis of 11 longitudinal studies showed that the deterioration in mental health during the first lockdown in the UK did not return to baseline levels when social restrictions were eased (76), hence increased psychological distress long-term seems to be a consequence of the pandemic (77). Our study suggests that there are no effects of psychological distress on changes over time in alcohol consumption. In addition, the rate of participants with psychological distress dropped from 20 to 16%.

The present study showed that older adults (50–70 years), compared with the ones below 30 years of age, reported higher alcohol consumption. This is consistent with previous general findings of a tendency for high-frequency drinking (5 or more days a week) to increase with age (78, 79). On the other hand, the alcohol consumption patterns of the youngest participants may be linked to social events such as weekend parties and nightlife, which could help explain the observed results. An increase in high-frequency drinking with age may be concerning from a public health perspective, as it could potentially contribute to adverse health outcomes among older adults. It may also pose a higher risk of medication-alcohol interactions, possibly compromising the safety and effectiveness of medications used by older adults.

It is also worrying that around 20% of men in the present sample display high and severe-risk drinking. The analysis demonstrates that men have a generally high alcohol consumption and a significantly higher one than women, which was sustained throughout the pandemic. Previous research indicates that people with high levels of psychological distress may resort to alcohol as a form of self-medication to cope with or escape from their symptoms (80, 81); however, this can have negative long-term effects, perpetuating a vicious cycle. This form of self-medication appears to apply particularly to men (44, 82). Men with higher distress levels generally report higher alcohol consumption (42, 43, 71). These gender differences have also been found outside the pandemic period in a comparable population (83).

Increased alcohol consumption is positively associated with elevated stress and anxiety levels (43). Stress and anxiety can be triggered by social isolation or quarantine (84). Studies have demonstrated that pandemic-induced stress can lead to elevated drinking levels and that alcohol consumption can function as a (maladaptive) coping strategy (85, 86). Boredom is also a crucial factor for increased alcohol consumption (87). Boredom and isolation, which are likely to occur to a greater extent during a pandemic, can cause distress (84). Both may have been exacerbated by reduced PA levels. This aligns with other studies that have reported that quarantine and social isolation were associated with reduced PA levels and that people who reported higher levels of stress during the pandemic were less likely to engage in PA (55, 88). A lack of motivation to engage in PA during the pandemic may be attributed to various factors, such as gym closures, limited opportunities to exercise outdoors, increased stress and anxiety levels, and depression symptoms (89, 90). The negative impact the pandemic has had on PA levels might in turn have contributed to increased stress levels in line with our findings.

Being laid off from work could have a negative impact on economic worries. Our results show that participants who were temporarily laid off at baseline did not report an increase in alcohol consumption at the latter measuring point. Compensation packages that the government introduced may have contributed to less economic worry and consequently less emotional/escapism drinking. Layoffs due to COVID-19 have now mostly been resolved.

Our findings revealed a significant correlation between working from home and increased alcohol intake as well as decreased PA. There is no significant change observed in either variable over time. Other studies have also shown an association between working from home and increased alcohol consumption and found similar patterns regarding psychological and socio-economic circumstances (91, 92). Therefore, the impact of working from home on behavioral risk factors should be taken into consideration as a public health concern and addressed accordingly.

In line with other studies (93–95), the results show that PA decreased during the first year of the COVID-19 pandemic. During the initial months of the pandemic, PA levels declined due to the lockdowns and social distancing orders that were imposed in many countries. A systematic review (31) reveals that over 50% of the examined population’s PA either stayed the same or decreased during lockdown. This trend is confirmed by a recent meta-analysis (96), demonstrating a declining trend of PA globally. The closure of gyms and other public spaces, as well as the fear of contracting the virus, most likely were contributing factors resulting in decreased PA levels.

In terms of PA levels, there were some differences based on gender, and differences were found among the age groups. Our findings showed that the oldest participants were generally less active than their younger peers, which is reasonable as they may have wanted to avoid the risk of infection. A recent systematic review showed a reduction in PA levels in the elderly worldwide attributed to the pandemic (57). At the same time, this is problematic because PA in older age decreases the risk of several lifestyle-related diseases and comorbidities (97). Lockdown periods during the pandemic may have limited PA opportunities. A certain reduction in PA 1 year into the pandemic was therefore expected. Furthermore, people had to work from home, thus, reducing PA created by commuting (e.g., cycling to work). All age groups showed a positive, albeit minimal, time trend over the 2 years. The PA levels in 2022 came back to the same levels as before the pandemic measures, after a short period of reduction in PA.

In addition to the physical restrictions, the mental and emotional toll of the pandemic can also have been a barrier to PA in terms of difficulty in finding the motivation to exercise while struggling with stress, anxiety, and depression. Fear of being infected, mental distress, and a weakened physical capacity because of a COVID-19 infection can explain decreased PA. Other studies identified being fearful of contamination with COVID-19 (98), and depressive symptoms (55) as the main barriers to engaging in PA. In line with our findings regarding psychological distress, this gives reason for concern as PA has been found to improve mental health outcomes for people with mental illness (99). Pandemic measures might create a vicious circle between PA and mental health disorders: limiting PA due to pandemic measures and, thus, dampening the beneficial effect PA has on mental health and weakening the motivation for PA because of worse mental health. We see an increase over time in PA among the participants who score high on psychological distress. Maintaining regular PA is therefore important to preserve mental health during societal lockdowns. In this realm, it should be noted that the closure of facilities and restrictions for PA might increase feelings of isolation, already intensified by lockdown or social distancing.

Overall, the findings suggest that measures to maintain PA during future potential lockdowns should be given priority by individuals, sports organizations, and health authorities, respectively.

### 4.1. Strengths and limitations

The present study had several strengths, including the ability to conduct highly precise and statistically powerful analyses due to the large sample size. Additionally, yearly follow-ups of the participants provided important insight into changes over time during the pandemic era. While a considerable proportion of participants dropped out during the follow-ups, we make adjustments that reduce the likelihood of substantial selection biases, and background factors in each of the groups were similar. On the other hand, the large sample size may result in findings that are statistically significant but not necessarily important and relevant differences. Even though the sample is randomly selected from a wide population, the differences in response between strata of the population may contribute to our cohort not necessarily being completely representative of the source population, with potential limitations in generalizability. Although the recruitment to the study was based on random sampling, the electronic approach could have influenced the results and could have limited people who are less digitally literate.

The level of PA was self-reported by participants, hence misclassification of people when using self-reported PA data is a risk (100) as people may not always accurately report their activity levels, leading to inaccurate results. The AUDIT-C also relies on self-reported information. Self-reported alcohol consumption often has an inherent limitation due to underreporting (101). Social desirability bias occurs when people answer questions in a way that will make them appear more socially desirable or accepted. Hence, people may overreport their PA level or downplay their alcohol consumption to provide answers that they think are more socially acceptable, even if they are inaccurate.

Moreover, the IPAQ-SF does not measure the intensity of PA but only measures its duration, which does not give an accurate picture of overall PA levels. Further, the IPAQ-SF does not account for different types of PA such as running and walking, which may have different health benefits. Overall, the IPAQ-SF is a valuable tool for measuring PA levels, but it has several inherent limitations that need to be considered (102). Yet, objective methods at the population level were impossible to implement during the pandemic.

Wintertime is also viewed as less favorable for outdoor activities (e.g., fewer hours of daylight, colder, more wind, and more rain) (103). Hence, the data collection period from mid-December to mid-January might have affected the responses regarding PA.

Furthermore, the AUDIT-C assesses self-reported information on alcohol consumption during the past 12 months. Thus, recall bias can significantly affect such information, as people’s memories may be inaccurate or incomplete. They might also give greater importance to recent events (recency bias) when providing responses (104).

## 5. Conclusion

In conclusion, participants with high levels of psychological distress were more likely to have higher alcohol consumption and lower levels of PA at the beginning of the pandemic. However, there was a positive trend to less difference in PA levels over time among those with high levels of psychological distress. Nevertheless, the pandemic may have an amplifying negative effect on those with certain (e.g., tendency to worry) characteristics.

## Author’s note

BiE study group: SM, LF, Stine Lehmann, Ragnhild Bjørknes, EH, William Hazell, Øystein Vedaa, JC, Gro Mjeldheim Sandal, Åsgeir Kjetland Rabben and Andreas Roaldsnes.

## Data availability statement

The datasets presented in this article are not readily available because of national regulations on health research. It can be available from the corresponding author on reasonable request. Requests to access the datasets should be directed to SA: silvia.eiken.alpers@helse-bergen.no.

## Ethics statement

The studies involving human participants were reviewed and approved by Regional Committee for Medical and Health Research Ethics, Western Norway. The participants had to tick a box in the questionnaire, to provide informed consent to participate in this study.

## Author contributions

SM and LF: conceptualization. SM, LF, JS, and SA: methodology. SA, JV, and LF: formal analysis. SA and LF: writing—original draft preparation. L-HL, SP, AM, JV, EH, and JS: writing—review and editing. LF: supervision. LF and SM: project administration. SA, SP, JV, EH, L-HL, JS, AM, SM, and LF contributed to interpretation and writing. All authors contributed to the article and approved the submitted version.

## Funding

This project was co-founded by the University of Bergen and Bergen municipality.

## Conflict of interest

The authors declare that the research was conducted in the absence of any commercial or financial relationships that could be construed as a potential conflict of interest.

## Publisher’s note

All claims expressed in this article are solely those of the authors and do not necessarily represent those of their affiliated organizations, or those of the publisher, the editors and the reviewers. Any product that may be evaluated in this article, or claim that may be made by its manufacturer, is not guaranteed or endorsed by the publisher.

## Supplementary material

The Supplementary material for this article can be found online at: https://www.frontiersin.org/articles/10.3389/fpsyt.2023.1181046/full#supplementary-material

## Footnotes

^1^The common contact register helps Norwegian public authorities to communicate digitally with citizens. The Norwegian Digitalization Agency is responsible for the contact information in the common contact register, which contains mobile phone numbers and e-mail addresses of citizens in Norway.

^2^ http://www.ipaq.ki.se

^3^ https://sankeymatic.com

## Abbreviations

AUDIT-C: alcohol use disorder identification test—consumption
CI: confidence interval
COVID-19: Coronavirus disease-2019
EUR: euro
IPAQ-SF: international physical activity questionnaire—short form
MET: metabolic equivalent of task
NOK: Norwegian krone
PA: physical activity
SCL-10: symptom checklist ten-items
