# Supplementary material for: The association between psychological distress and alcohol consumption and physical activity: a population-based cohort study
Source: Front Psychiatry. 2023 Jun 22;14:1181046. doi: 10.3389/fpsyt.2023.1181046 (PMC10323831; doi:10.3389/fpsyt.2023.1181046)
Supplement: Supplementary file 1 [file Data_Sheet_1.docx]

Supplementary Material

# Supplementary Figures and Tables

## Supplementary Tables

**Supplementary Table 1.** Background characteristics of participants per age group, April 2020

| **Age** | **18–29** | **30–39** | **40–49** | **50–59** | **60–69** | **70+** | **Total**  ***n* (%)** |
| --- | --- | --- | --- | --- | --- | --- | --- |
| ***n*** | 3347 (13%) | 4167 (16%) | 4733 (18%) | 5292 (21%) | 4510 (18%) | 3659 (14%) | 25,708 (100%) |
| Gender (women) | 2166 (65%) | 2501 (60%) | 2738 (58%) | 2989 (56%) | 2318 (51%) | 1740 (48%) | 14,452 (56%) |
| Primary school | 424 (13%) | 157 (4%) | 188 (4%) | 282 (5%) | 399 (9%) | 474 (13%) | 1924 (8%) |
| High school | 1196 (36%) | 787 (19%) | 952 (20%) | 1647 (31%) | 1463 (33%) | 1201 (33%) | 7246 (28%) |
| University ≤ 3 years | 871 (26%) | 1067 (26%) | 1128 (24%) | 1277 (24%) | 1000 (22%) | 814 (23%) | 6157 (24%) |
| University > 3 years | 844 (25%) | 2132 (51%) | 2448 (52%) | 2064 (39%) | 1630 (36%) | 1128 (31%) | 10,246 (40%) |
| Adjusted income (EUR) * |  |  |  |  |  |  |  |
| 0–25,000 | 1054 (36%) | 537 (13%) | 478 (11%) | 377 (8%) | 251 (7%) | 383 (13%) | 3080 (13%) |
| 25,000–50,000 | 1116 (38%) | 1977 (50%) | 2301 (51%) | 1839 (38%) | 1380 (36%) | 1438 (50%) | 10,051 (44%) |
| >50,000 | 739 (25%) | 1474 (37%) | 1700 (38%) | 2596 (54%) | 2229 (58%) | 1051 (37%) | 9789 (43%) |
| Persons in household |  |  |  |  |  |  |  |
| 1 | 477 (14%) | 617 (15%) | 514 (11%) | 880 (17%) | 1234 (29%) | 1460 (43%) | 5182 (21%) |
| 2 | 1176 (36%) | 886 (22%) | 615 (13%) | 1618 (31%) | 2208 (51%) | 1554 (46%) | 8057 (32%) |
| 3–4 | 1223 (37%) | 1964 (48%) | 2314 (50%) | 2144 (42%) | 800 (18%) | 331 (10%) | 8776 (35%) |
| 5+ | 433 (13%) | 649 (16%) | 1210 (26%) | 510 (10%) | 83 (2%) | 50 (1%) | 2935 (12%) |
| Employment | 2206 (66%) | 3597 (86%) | 4241 (90%) | 4650 (88%) | 2523 (56%) | 230 (6%) | 17,447 (68%) |
| Student/school | 1607 (48%) | 257 (6%) | 105 (2%) | 32 (1%) | 7 (0%) | 3 (0%) | 2011 (8%) |
| Placed in quarantine | 747 (22%) | 681 (16%) | 716 (15%) | 744 (14%) | 682 (15%) | 603 (16%) | 4173 (16%) |
| Temporarily laid-off | 513 (15%) | 401 (10%) | 362 (8%) | 419 (8%) | 225 (5%) | 20 (1%) | 1940 (8%) |
| Home office/study | 2217 (66%) | 2711 (65%) | 3179 (67%) | 2909 (55%) | 1476 (33%) | 154 (4%) | 12,646 (49%) |
| COVID-19 symptoms | 279 (8%) | 353 (8%) | 376 (8%) | 328 (6%) | 165 (4%) | 80 (2%) | 1581 (6%) |
| Worries | 2209 (66%) | 2499 (60%) | 2510 (53%) | 2861 (54%) | 1857 (41%) | 1145 (31%) | 13,081 (51%) |
| Worries related to economy | 978 (29%) | 1009 (24%) | 881 (19%) | 866 (16%) | 364 (8%) | 81 (2%) | 4179 (16%) |
| Health-related worries | 1850 (55%) | 2051 (49%) | 2165 (46%) | 2518 (48%) | 1684 (37%) | 1099 (30%) | 11,367 (44%) |
| Psychological distress | 1359 (41%) | 1175 (28%) | 912 (19%) | 813 (15%) | 500 (11%) | 293 (8%) | 5052 (20%) |

* The adjusted income is the household income divided by the personal index. The personal index is calculated as 1 for the first adult, 0.7 per other adult household member, and 0.5 per child. The adjusted income was converted to Euros.

**Supplementary Table 2.** Background characteristics of participants per age group, January 2021

| **Age** | **18–29** | **30–39** | **40–49** | **50–59** | **60–69** | **70+** | **Total**  ***n* (%)** |
| --- | --- | --- | --- | --- | --- | --- | --- |
| ***n*** | 1641 (9%) | 2512 (15%) | 3134 (18%) | 3773 (22%) | 3494 (20%) | 2738 (16%) | 17,292 (100%) |
| Gender (women) | 1068 (65%) | 1538 (61%) | 1871 (60%) | 2191 (58%) | 1780 (51%) | 1301 (48%) | 9749 (56%) |
| Primary school | 169 (12%) | 70 (3%) | 102 (3%) | 167 (5%) | 269 (8%) | 296 (11%) | 1073 (7%) |
| High school | 462 (32%) | 371 (16%) | 543 (19%) | 1107 (31%) | 1072 (32%) | 849 (32%) | 4404 (27%) |
| University ≤ 3 years | 393 (27%) | 589 (26%) | 687 (24%) | 885 (25%) | 779 (23%) | 617 (23%) | 3950 (24%) |
| University > 3 years | 411 (29%) | 1280 (55%) | 1591 (54%) | 1451 (40%) | 1256 (37%) | 885 (33%) | 6874 (42%) |
| Adjusted income (EUR) * |  |  |  |  |  |  |  |
| 0–25,000 | 422 (33%) | 229 (10%) | 260 (9%) | 247 (7%) | 176 (6%) | 255 (12%) | 1589 (11%) |
| 25,000–50,000 | 475 (37%) | 1077 (48%) | 1430 (51%) | 1240 (37%) | 1041 (35%) | 1083 (50%) | 6346 (43%) |
| >50,000 | 372 (29%) | 935 (42%) | 1112 (40%) | 1840 (55%) | 1729 (59%) | 809 (38%) | 6797 (46%) |
| Persons in household |  |  |  |  |  |  |  |
| 1 | 224 (16%) | 377 (16%) | 340 (12%) | 634 (18%) | 897 (27%) | 1041 (42%) | 3513 (22%) |
| 2 | 524 (37%) | 497 (22%) | 386 (13%) | 1130 (32%) | 1709 (52%) | 1190 (48%) | 5436 (34%) |
| 3–4 | 500 (35%) | 1066 (46%) | 1428 (49%) | 1425 (40%) | 605 (18%) | 241 (10%) | 5265 (33%) |
| 5+ | 174 (12%) | 357 (16%) | 737 (25%) | 348 (10%) | 61 (2%) | 31 (1%) | 1708 (11%) |
| Employment | 990 (60%) | 2045 (81%) | 2662 (85%) | 3195 (85%) | 1881 (54%) | 166 (6%) | 10,939 (63%) |
| Student/school | 644 (39%) | 105 (4%) | 65 (2%) | 20 (1%) | 4 (0%) | 2 (0%) | 840 (5%) |
| Placed in quarantine | 357 (22%) | 415 (17%) | 479 (15%) | 541 (14%) | 532 (15%) | 484 (18%) | 2808 (16%) |
| Temporarily laid-off | 210 (13%) | 186 (7%) | 211 (7%) | 289 (8) | 166 (5%) | 14 (1%) | 1076 (6%) |
| Home office/study | 1085 (66%) | 1671 (67%) | 2132 (68%) | 2122 (56%) | 1145 (33%) | 121 (4%) | 8276 (48%) |
| COVID-19 symptoms | 131 (8%) | 210 (8%) | 244 (8%) | 231 (6%) | 118 (3%) | 51 (2%) | 985 (6%) |
| Worries | 931 (57%) | 1380 (55%) | 1525 (49%) | 1949 (52%) | 1373 (39%) | 806 (29%) | 7964 (46%) |
| Worries related to economy | 388 (24%) | 519 (21%) | 491 (16%) | 574 (15%) | 264 (8%) | 63 (2%) | 2299 (13%) |
| Health-related worries | 788 (48%) | 1162 (46%) | 1317 (42%) | 1703 (45%) | 1243 (36%) | 768 (28%) | 6981 (40%) |
| Psychological distress | 576 (35%) | 673 (27%) | 552 (18%) | 544 (14%) | 358 (10%) | 185 (7%) | 2888 (17%) |

* The adjusted income is the household income divided by the personal index. The personal index is calculated as 1 for the first adult, 0.7 per other adult household member, and 0.5 per child. The adjusted income was converted to Euros.

**Supplementary Table 3.** Background characteristics of participants per age group, January 2022

| **Age** | **18–29** | **30–39** | **40–49** | **50–59** | **60–69** | **70+** | **Total**  ***n* (%)** |
| --- | --- | --- | --- | --- | --- | --- | --- |
| ***n*** | 807 (8%) | 1359 (13%) | 1752 (17%) | 2343 (23%) | 2265 (22%) | 1730 (17%) | 10,256 (100%) |
| Gender (women) | 554 (69%) | 866 (64%) | 1089 (62%) | 1379 (59%) | 1161 (51%) | 837 (48%) | 5886 (57%) |
| Primary school | 85 (12%) | 36 (3%) | 50 (3%) | 101 (4%) | 163 (7%) | 163 (10%) | 598 (6%) |
| High school | 226 (31%) | 204 (16%) | 293 (18%) | 639 (28%) | 652 (30%) | 528 (31%) | 2542 (26%) |
| University ≤ 3 years | 205 (28%) | 305 (24%) | 389 (24%) | 534 (24%) | 492 (22%) | 407 (24%) | 2332 (24%) |
| University > 3 years | 222 (30%) | 715 (57%) | 923 (56%) | 980 (43%) | 891 (41%) | 584 (35%) | 4315 (44%) |
| Adjusted income (EUR) * |  |  |  |  |  |  |  |
| 0–25,000 | 210 (33%) | 131 (11%) | 139 (9%) | 143 (7%) | 101 (5%) | 153 (11%) | 877 (10%) |
| 25,000–50,000 | 261 (40%) | 581 (48%) | 824 (52%) | 759 (36%) | 680 (35%) | 694 (50%) | 3799 (43%) |
| >50,000 | 175 (27%) | 508 (42%) | 625 (39%) | 1197 (57%) | 1151 (60%) | 543 (39%) | 4199 (47%) |
| Persons in household |  |  |  |  |  |  |  |
| 1 | 129 (18%) | 216 (17%) | 200 (12%) | 417 (19%) | 568 (27%) | 656 (41%) | 2186 (23%) |
| 2 | 285 (39%) | 273 (22%) | 225 (14%) | 708 (32%) | 1130 (53%) | 756 (47%) | 3377 (35%) |
| 3–4 | 228 (31%) | 589 (47%) | 795 (49%) | 888 (40%) | 405 (19%) | 172 (11%) | 3077 (32%) |
| 5+ | 85 (12%) | 173 (14%) | 418 (26%) | 198 (9%) | 36 (2%) | 24 (1%) | 934 (10%) |
| Employment | 508 (63%) | 1119 (82%) | 1503 (86%) | 2008 (86%) | 1230 (54%) | 100 (6%) | 6468 (63%) |
| Student/school | 331 (41%) | 68 (5%) | 39 (2%) | 14 (1%) | 2 (0%) | 2 (0%) | 456 (4%) |
| Placed in quarantine | 167 (21%) | 208 (16%) | 270 (15%) | 347 (15%) | 353 (16%) | 281 (16%) | 1626 (16%) |
| Temporarily laid-off | 105 (13%) | 96 (7%) | 96 (5%) | 170 (7%) | 109 (5%) | 5 (0%) | 581 (6%) |
| Home office/study | 551 (68%) | 900 (66%) | 1188 (68%) | 1352 (58%) | 768 (34%) | 73 (4%) | 4832 (47%) |
| COVID-19 symptoms | 64 (8%) | 114 (8%) | 143 (8%) | 147 (6%) | 89 (4%) | 37 (2%) | 594 (6%) |
| Worries | 470 (58%) | 718 (53%) | 850 (49%) | 1203 (51%) | 885 (39%) | 483 (28%) | 4609 (45%) |
| Worries related to economy | 197 (24%) | 277 (20%) | 267 (15%) | 326 (14%) | 172 (8%) | 33 (2%) | 1271 (12%) |
| Health-related worries | 388 (48%) | 584 (43%) | 746 (43%) | 1073 (46%) | 798 (35%) | 462 (27%) | 4051 (40%) |
| Psychological distress | 288 (36%) | 362 (27%) | 315 (18) | 344 (15%) | 249 (11%) | 114 (7%) | 1672 (16%) |

* The adjusted income is the household income divided by the personal index. The personal index is calculated as 1 for the first adult, 0.7 per other adult household member, and 0.5 per child. The adjusted income was converted to Euros.

**Supplementary Table 4.** Drinking behavior and PA levels in relation to age for women. Baseline, 1-year follow-up, and 2-year follow-up (n (%))

| Age | 18-29 | 30-39 | 40-49 | 50-59 | 60-69 | 70+ | Total |
| --- | --- | --- | --- | --- | --- | --- | --- |
| BASELINE |  |  |  |  |  |  |  |
| Low-risk drinking* | 655 (30%) | 1,150 (46%) | 1,212 (44%) | 1,368 (46%) | 1,106 (48%) | 1,036 (62%) | 6,527 (46%) |
| Moderate-risk drinking | 1,058 (49%) | 1,146 (46%) | 1,342 (49%) | 1,416 (48%) | 1,061 (46%) | 601 (36%) | 6,624 (46%) |
| High-risk drinking | 368 (17%) | 166 (7%) | 141 (5%) | 160 (5%) | 106 (5%) | 41 (2%) | 982 (7%) |
| Severe-risk drinking | 77 (4%) | 32 (1%) | 32 (1%) | 27 (1%) | 24 (1%) | 6 (0%) | 198 (1%) |
| PA level low | 573 (27%) | 689 (29%) | 656 (26%) | 598 (22%) | 372 (20%) | 328 (28%) | 3216 (25%) |
| PA level moderate | 957 (45%) | 1106 (46%) | 1189 (46%) | 1284 (48%) | 917 (48%) | 530 (45%) | 5983 (47%) |
| PA level high | 580 (27%) | 611 (25%) | 714 (28%) | 799 (30%) | 605 (32%) | 314 (27%) | 3623 (28%) |
| 1-YEAR FOLLOW-UP |  |  |  |  |  |  |  |
| Low-risk drinking | 283 (30%) | 612 (43%) | 749 (43%) | 973 (46%) | 808 (47%) | 732 (59%) | 4,157 (45%) |
| Moderate-risk drinking | 487 (52%) | 681 (48%) | 904 (51%) | 1,003 (48%) | 820 (48%) | 468 (38%) | 4,363 (47%) |
| High-risk drinking | 147 (16%) | 105 (7%) | 83 (5%) | 111 (5%) | 85 (5%) | 31 (3%) | 562 (6%) |
| Severe-risk drinking | 28 (3%) | 20 (1%) | 20 (1%) | 21 (1%) | 13 (1%) | 6 (0%) | 108 (1%) |
| PA level low | 257 (27%) | 411 (29%) | 425 (25%) | 428 (22%) | 274 (19%) | 225 (25%) | 2020 (24%) |
| PA level moderate | 450 (47%) | 673 (47%) | 798 (47%) | 933 (48%) | 725 (49%) | 422 (47%) | 4001 (48%) |
| PA level high | 250 (26%) | 346 (24%) | 486 (28%) | 579 (30%) | 468 (32%) | 247 (28%) | 2376 (28%) |
| 2-YEAR FOLLOW-UP |  |  |  |  |  |  |  |
| Low-risk drinking | 145 (29%) | 345 (43%) | 439 (43%) | 614 (46%) | 537 (48%) | 462 (57%) | 2,542 (45%) |
| Moderate-risk drinking | 270 (53%) | 394 (49%) | 525 (51%) | 634 (47%) | 529 (47%) | 316 (39%) | 2,668 (48%) |
| High-risk drinking | 72 (14%) | 58 (7%) | 56 (5%) | 77 (6%) | 55 (5%) | 26 (3%) | 344 (6%) |
| Severe-risk drinking | 18 (4%) | 10 (1%) | 11 (1%) | 12 (1%) | 6 (1%) | 3 (0%) | 60 (1%) |
| PA level low | 144 (28%) | 221 (27%) | 258 (26%) | 264 (21%) | 176 (18%) | 145 (24%) | 1208 (23%) |
| PA level moderate | 242 (47%) | 401 (50%) | 487 (48%) | 617 (50%) | 477 (49%) | 288 (47%) | 2512 (49%) |
| PA level high | 125 (24%) | 186 (23%) | 266 (26) | 365 (29%) | 311 (32%) | 175 (29%) | 1428 (28%) |

* Drinking categories are based on AUDIT-C scores.

The table displays population-weighted estimates (age, gender, education) for percentages.

**Supplementary Table 5.** Drinking behavior and PA levels in relation to age for men. Baseline, 1-year follow-up, and 2-year follow-up (n (%))

| Age | 18-29 | 30-39 | 40-49 | 50-59 | 60-69 | 70+ | Total |
| --- | --- | --- | --- | --- | --- | --- | --- |
| BASELINE |  |  |  |  |  |  |  |
| Low-risk drinking* | 409 (35%) | 750 (45%) | 867 (44%) | 1,042 (45%) | 1,002 (46%) | 1,233 (65%) | 5,303 (47%) |
| Moderate-risk drinking | 348 (29%) | 566 (34%) | 731 (37%) | 793 (35%) | 765 (35%) | 497 (26%) | 3,700 (33%) |
| High-risk drinking | 290 (25%) | 241 (15%) | 257 (13%) | 329 (14%) | 297 (14%) | 121 (6%) | 1,535 (14%) |
| Severe-risk drinking | 133 (11%) | 99 (6%) | 129 (7%) | 130 (6%) | 118 (5%) | 44 (2%) | 653 (6%) |
| PA level low | 360 (31%) | 467 (29%) | 495 (26%) | 469 (22%) | 436 (22%) | 379 (26%) | 2606 (26%) |
| PA level moderate | 420 (37%) | 621 (38%) | 742 (40%) | 832 (39%) | 766 (39%) | 620 (43%) | 4001 (39%) |
| PA level high | 364 (32%) | 529 (33%) | 641 (34%) | 809 (38%) | 741 (38%) | 458 (31%) | 3542 (35%) |
| 1-YEAR FOLLOW-UP |  |  |  |  |  |  |  |
| Low-risk drinking | 171 (35%) | 398 (45%) | 495 (43%) | 667 (45%) | 747 (46%) | 876 (64%) | 3,354 (48%) |
| Moderate-risk drinking | 146 (30%) | 309 (35%) | 444 (38%) | 535 (36%) | 585 (36%) | 372 (27%) | 2,391 (34%) |
| High-risk drinking | 113 (23%) | 131 (15%) | 152 (13%) | 216 (14%) | 224 (14%) | 93 (7%) | 929 (13%) |
| Severe-risk drinking | 58 (12%) | 56 (6%) | 68 (6%) | 79 (5%) | 84 (5%) | 32 (2%) | 377 (5%) |
| PA level low | 165 (34%) | 255 (28%) | 304 (26%) | 301 (21%) | 315 (21%) | 252 (23%) | 1592 (24%) |
| PA level moderate | 179 (36%) | 357 (39%) | 477 (41%) | 584 (41%) | 614 (41%) | 489 (45%) | 2700 (41%) |
| PA level high | 148 (30%) | 295 (33%) | 369 (32%) | 536 (38%) | 571 (38%) | 352 (32%) | 2271 (35%) |
| 2-YEAR FOLLOW-UP |  |  |  |  |  |  |  |
| Low-risk drinking | 83 (36%) | 217 (48%) | 273 (44%) | 418 (46%) | 483 (45%) | 540 (63%) | 2,014 (49%) |
| Moderate-risk drinking | 62 (27%) | 141 (31%) | 224 (36%) | 318 (35%) | 392 (37%) | 233 (27%) | 1,370 (33%) |
| High-risk drinking | 63 (27%) | 64 (14%) | 80 (13%) | 128 (14%) | 146 (14%) | 63 (7%) | 544 (13%) |
| Severe-risk drinking | 24 (10%) | 31 (7%) | 44 (7%) | 48 (5%) | 45 (4%) | 24 (3%) | 216 (5%) |
| PA level low | 83 (36%) | 136 (29%) | 182 (30%) | 189 (22%) | 211 (21%) | 164 (23%) | 965 (25%) |
| PA level moderate | 87 (38%) | 172 (37%) | 223 (37%) | 354 (41%) | 407 (41%) | 321 (46%) | 1564 (40%) |
| PA level high | 60 (26%) | 155 (33%) | 206 (34%) | 328 (38%) | 377 (38%) | 217 (31%) | 1343 (35%) |

* Drinking categories are based on AUDIT-C scores.

The table displays population-weighted estimates (age, gender, education) for percentages.

## Supplementary Figures

**
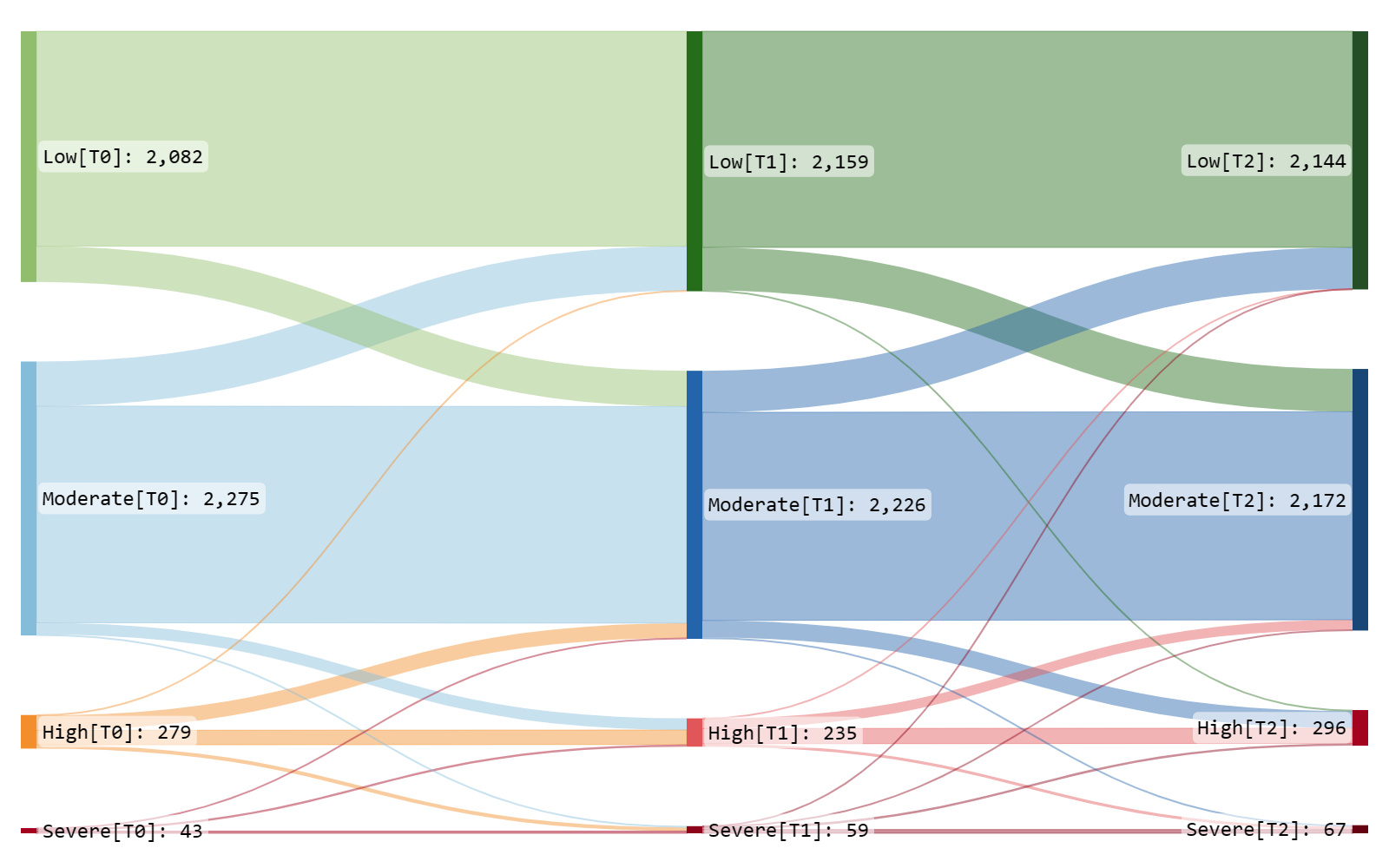
**

**Supplementary Figure 1.** Sankey diagram of change in alcohol consumption per gender, women (Low = AUDIT-C score ≤2 for women and ≤3 for men, Moderate = AUDIT-C score >2 and ≤5 for women and >3 and ≤5 for men, High = AUDIT-C score >5 and ≤7 for women and men, Severe = AUDIT-C score ≥8 for women and men)

**
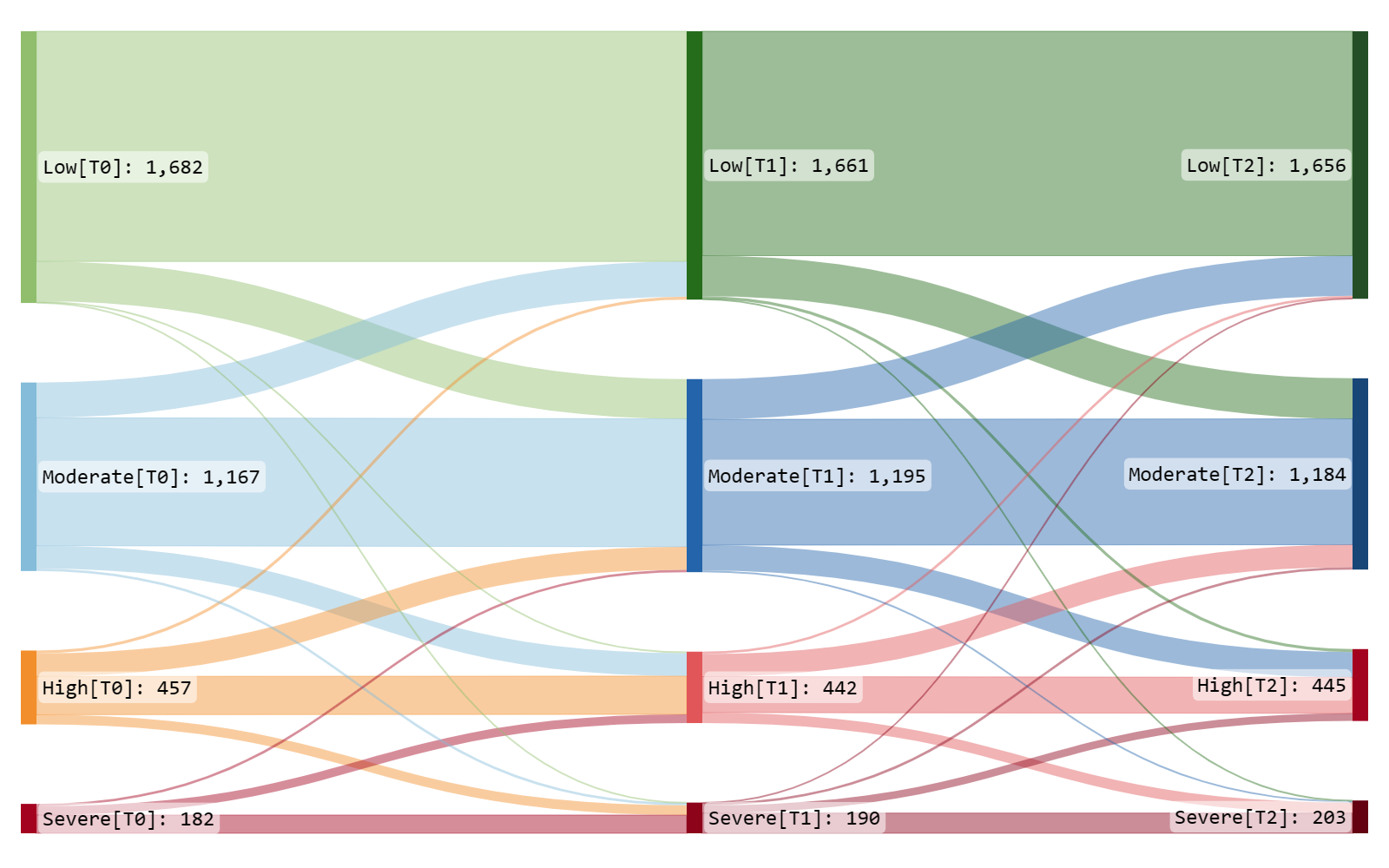
**

**Supplementary Figure 2.** Sankey diagram of change in alcohol consumption per gender, men (Low = AUDIT-C score ≤2 for women and ≤3 for men, Moderate = AUDIT-C score >2 and ≤5 for women and >3 and ≤5 for men, High = AUDIT-C score >5 and ≤7 for women and men, Severe = AUDIT-C score ≥8 for women and men)

**
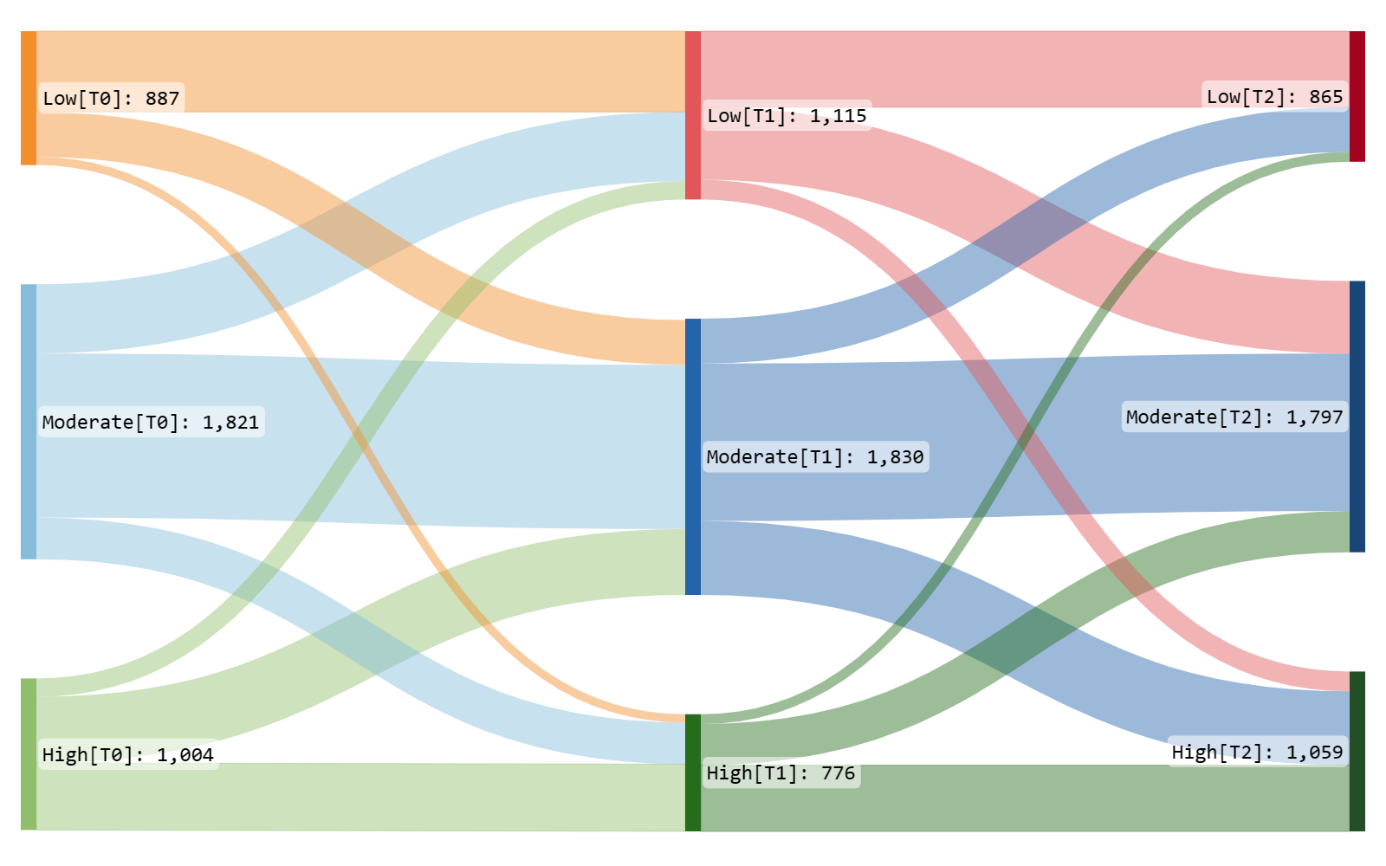
**

**Supplementary Figure 3.** Sankey diagram of change in physical activity per gender, women (High = >1 hour of moderate-intensity activity over and above basal activity or >30 minutes of vigorous-intensity activity above basal levels daily, Moderate = 30 minutes of at least moderate-intensity activity on most days of the week, Low = not meeting the aforementioned criteria)

**
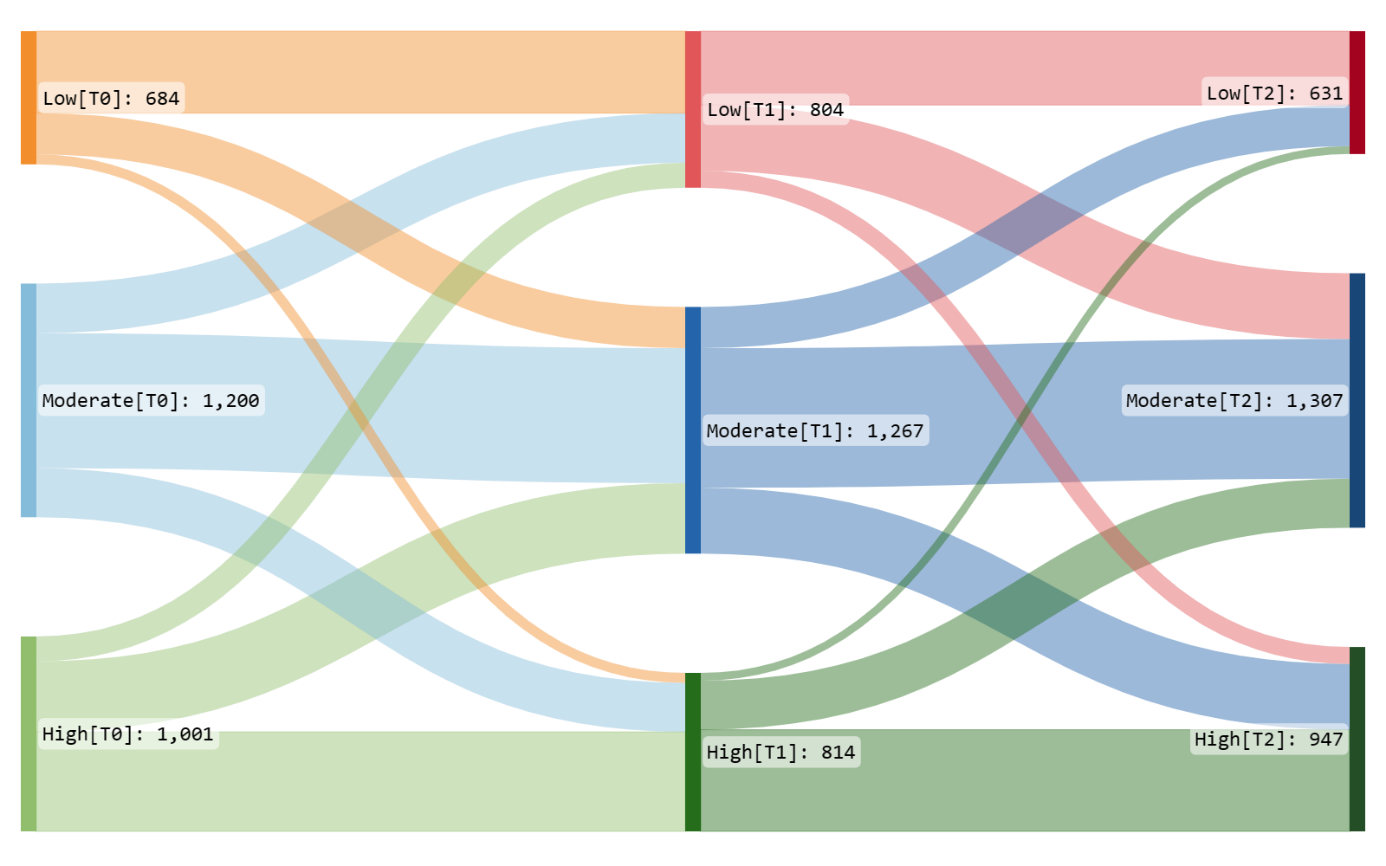
**

**Supplementary Figure 4.** Sankey diagram of change in physical activity per gender, men (High = >1 hour of moderate-intensity activity over and above basal activity or >30 minutes of vigorous-intensity activity above basal levels daily, Moderate = 30 minutes of at least moderate-intensity activity on most days of the week, Low = not meeting the aforementioned criteria)
